# The Health‐Related Quality of Life for Cemented Versus Uncemented Hemiarthroplasty in Elderly Patients With Femoral Neck Fractures: A Systematic Review and Meta‐Analysis of Randomized Controlled Trials

**DOI:** 10.1111/os.14339

**Published:** 2024-12-26

**Authors:** Mohanad Samaheen, Maen Mohammad, Mikhail Salzmann, Nikolai Ramadanov

**Affiliations:** ^1^ Faculty of Medicine Al‐Quds University Jerusalem Palestine; ^2^ Medical School Brandenburg Theodor Fontane University Hospital Brandenburg Brandenburg an der Havel Germany

**Keywords:** cemented, health‐related quality of life, hemiarthroplasty, hip fractures, uncemented

## Abstract

**Objective:**

Femoral neck fractures in the elderly are a global health issue, with the choice between cemented and uncemented hemiarthroplasty remaining a topic of debate. This systematic review and meta‐analysis aims to compare the effects of the two surgical options on health‐related quality of life (HRQoL), mortality, and functional outcomes.

**Methods:**

We searched PubMed, Embase, and Cochrane databases for randomized controlled trials (RCTs) comparing cemented with uncemented hemiarthroplasty in patients aged 50 years and older with femoral neck fractures. The primary outcome of interest was HRQoL as measured by the European Quality of Life 5‐Dimension Questionnaire (EQ‐5D) score. Secondary outcomes included mortality, surgical, general, and local complications.

**Results:**

We included 20 RCTs with a total of 3680 patients with femoral neck fractures, of whom 1871 (50.5%) underwent cemented and 1809 (49.5%) uncemented hemiarthroplasty. The follow‐up ranged from 1 to 6 years. The early (after 3–4 months) EQ‐5D utility score (MD 0.07; 95% CI 0.03–0.12; *p* = 0.003; *I* ^2^ = 22%) and the 12‐month EQ‐5D utility score (MD 0.08; 95% CI 0.00–0.16; *p* = 0.04; *I* ^2^ = 67%) suggested an improved HRQoL in the cemented hemiarthroplasty group. The outcomes of 1‐year mortality, requirement for additional surgeries, surgery duration, risk of pulmonary embolism, pressure sores or ulcers, intraoperative fractures, and periprosthetic or postoperative fractures demonstrated significant differences between the two groups.

**Conclusions:**

The use of cemented hemiarthroplasty in patients with femoral neck fractures presented better results when compared to uncemented hemiarthroplasty in terms of HRQoL during the first year after surgery and greater mortality reduction at 1 year follow‐up and reduced the need for further surgery. Therefore, the use of cemented hemiarthroplasty may be preferred for the treatment of femoral neck fractures in elderly patients.

## Graphical abstract

Cemented hemiarthroplasty improves health‐related quality of life, reduces mortality during the first year, and reduces the need for further surgical interventions compared to uncemented hemiarthroplasty in elderly patients with femoral neck fractures.

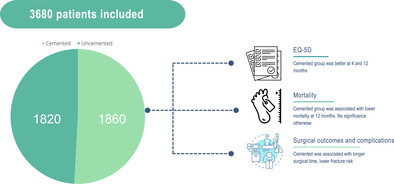

## 1 Introduction

Hip fractures in elderly individuals pose a growing global health threat, impacting an estimated one in four individuals over the age of 75. By 2050, the incidence is projected to exceed 7 million annually [1, 2]. Beyond the immediate health risks, these fractures often lead to disability, loss of independence, and increased care and support needs, placing significant financial strain on healthcare systems and compromising the quality of life of millions of people [3, 4].

The management of hip fractures, particularly of the femoral neck, which accounts for approximately half of all hip fractures, requires careful consideration of several factors including the patient's age, health, and activity level [5]. Two main surgical options are available for treating displaced femoral neck fractures (FNFs): hip hemiarthroplasty (HA) and total hip replacement (THR). The hip joint is a ball‐and‐socket joint, with the femoral head representing the ball and the acetabulum representing the socket. HA replaces the femoral head with an artificial “half‐joint” that articulates with the natural acetabulum in the pelvis and a stem that goes into the femur. However, in THR, the acetabulum is also replaced. HA is often preferred for elderly, frail, or low‐mobility patients who may be at greater risk of complications from more extensive surgery like THR [6, 7, 8].

There are generally two types of hip HA depending on the method of fixation used: cemented and uncemented HA. In cemented implants, bone cement fixates the implant inside the femur. Polymethylmethacrylate (PMMA), which is a type of bone cement, is considered the most widely used material in hip replacement surgery [9]. The implant is not fixated in uncemented hemiarthroplasties like in the cemented ones. The prosthesis coating is porous, allowing for a direct connection between the bone and the artificial implant. This promotes osseointegration, fixation, and bone ingrowth into tiny holes, providing more natural fixation with the implant. One of the most common materials used for uncemented implants is hydroxyapatite coating. Uncemented implants can be fully coated or partially coated [10].

The optimal fixation method for HA implants is controversial [11, 12]. A Cochrane review and meta‐analysis revealed that cemented implants were associated with less pain and greater mobility compared to early “press‐fit” uncemented designs [13]. However, cement injection during surgery carries potential risks including bone cement implantation syndrome (BCIS) manifesting as transient blood pressure drops and, in rare cases, cardiovascular collapse [14]. Newer hydroxyapatite‐coated uncemented implants seek to address these concerns by promoting improved bone integration, potentially leading to reliable fixation and a faster return to normal activities.

While a number of meta‐analyses have compared clinical outcomes [7, 15, 16, 17, 18, 19, 20], such as pain levels, mobility, and complications, a crucial aspect remains largely unexplored: the impact on patients' health‐related quality of life (HRQoL). Because patients with FNFs typically endure pain and marked functional impairment, prompt and effective therapeutic intervention is crucial in the management of those fractures. This meta‐analysis addresses this critical knowledge gap by focusing on the differences in HRQoL outcomes, such as the European Quality of Life 5‐Dimension Questionnaire (EQ‐5D) score in patients undergoing these procedures. Moreover, we will look at the differences in mortality rates, surgical complications, and local complications between the two groups. Ultimately, the aim is to guide clinicians toward a more patient‐centered approach that prioritizes physical recovery and considers the crucial impact on patients' emotional, social, and overall well‐being.

## 2 Methods

### 2.1 Eligibility Criteria

In this meta‐analysis, we included only randomized controlled trials (RCTs) that (1) compared cemented to uncemented HA, (2) included patients aged more than 50 years with FNFs, (3) had a minimum follow‐up period of 3 months, and (4) reported any of the clinical outcomes of interest.

We excluded studies (1) with no control group, (2) that were abstracts, comments, nonrandomized clinical trials, case reports, reviews, literature reviews, or previous meta‐analyses or for which data could not be extracted or estimated, (3) that were published in any language other than English, (4) that were conducted on animals or cadavers, and (5) that failed to report results relevant to the outcomes of interest.

### 2.2 Registration and Search Strategy

We employed a systematic review methodology aligned with the Preferred Reporting Items for Systematic Reviews and Meta‐Analyses (PRISMA) guidelines [21]. The review protocol was registered in the PROSPERO international prospective register of systematic reviews database under registration number (CRD42023486005).

Two reviewers, M.S. and M.M., independently searched the PubMed, EMBASE, and Cochrane Library databases, covering literature published up to November 30, 2023. The search strategy involved a comprehensive array of terms related to the condition, as detailed in the Supporting Information: Appendix S1. Additionally, a manual search was conducted through the references of all included studies as well as previous systematic reviews and meta‐analyses to identify any additional studies.

### 2.3 Literature Screening

Two researchers, M.S. and M.M., independently executed the screening process using predefined eligibility criteria and utilized Rayyan software to eliminate duplicate studies. Numerous studies were excluded after their titles and abstracts were evaluated. The full texts of the remaining studies were meticulously reviewed to ascertain their adherence to the inclusion criteria. The selected studies were subsequently cross‐verified, with any disagreements resolved through discussion. In cases where a consensus was not reached, the final decision was deferred to the senior author, N.R.

### 2.4 Data Extraction

Data extraction was performed utilizing a predesigned table. Two authors (M.S. and M.M.) independently extracted key study characteristics. These included details such as the first author's name, publication date, country of study, study design, sample size, average participant age, sex distribution, intervention and control used, prosthesis specifications, American Society of Anesthesiologists (ASA) grade scale, duration of the follow‐up period, and pertinent clinical outcomes.

### 2.5 Outcomes of Interest and Definitions

We included studies that reported any of the outcomes of interest. The primary outcome of our study was HRQoL, as measured by the EQ‐5D, a standardized HRQoL instrument developed by the EuroQol Group. It is a generic measure for assessing health outcomes in clinical and economic appraisals. It evaluates five dimensions, namely, mobility, self‐care, usual activities, pain/discomfort, and anxiety/depression, which collectively afford a comprehensive assessment of patients' health status pre‐ and post‐surgery.

Secondary outcomes of interest included (1) mortality rates (within 4 months, at 1 year, and at 2 years), (2) surgical‐related outcomes (need for additional surgery, length of surgery, duration of hospital stay, intraoperative blood loss, required for blood transfusion, and units of blood transfused), (3) general complications (pulmonary infections, cardiovascular events, deep vein thrombosis (DVT), pulmonary embolism (PE), pressure sores/ulcer/decubitus, cerebrovascular accident, acute renal failure, and urinary tract infections (UTIs)), and (4) local complications (dislocation, intraoperative fracture, periprosthetic/postoperative fracture, wound hematoma, superficial infection, and deep infection). The need for additional surgeries refers to any subsequent surgical procedures required after the initial intervention. This encompasses revisions, reoperations, or other interventions deemed necessary to manage complications or ensure the primary surgery's success.

### 2.6 Quality Assessment

Risk of bias assessment was conducted using the Cochrane risk‐of‐bias tool for RCTs [22]. Selection, performance, detection, attrition, and reporting biases were evaluated as high, low, or unclear. Disagreements were resolved through consensus. Publication bias was assessed by visual inspection of funnel plots and Egger's test.

### 2.7 Statistical Analysis

We calculated the risk ratio (RR) using a 95% confidence interval (CI) for dichotomous outcomes and the mean difference (MD) with a 95% CI for continuous outcomes. The heterogeneity between the included studies was tested using both the chi‐square (*χ* ^2^) test and I‐square (*I* ^2^) test, and the *p*‐value was > 0.10. Heterogeneity was defined as absent (*I* ^2^ = 0.0%–25.0%), low (*I* ^2^ = 25.1%–50.0%), moderate (*I* ^2^ = 50.1%–75.0%), or high (*I* ^2^ = 75.1%–100.0%). A random‐effects model was used to minimize the effect of heterogeneity. The result was considered significant for a probability value (*p*) of < 0.05. All the statistical analyses adhered to the Cochrane recommendations and were conducted using Review Manager 5.4.1 software.

## 3 Results

### 3.1 Study Selection and Characteristics

**FIGURE 1:**
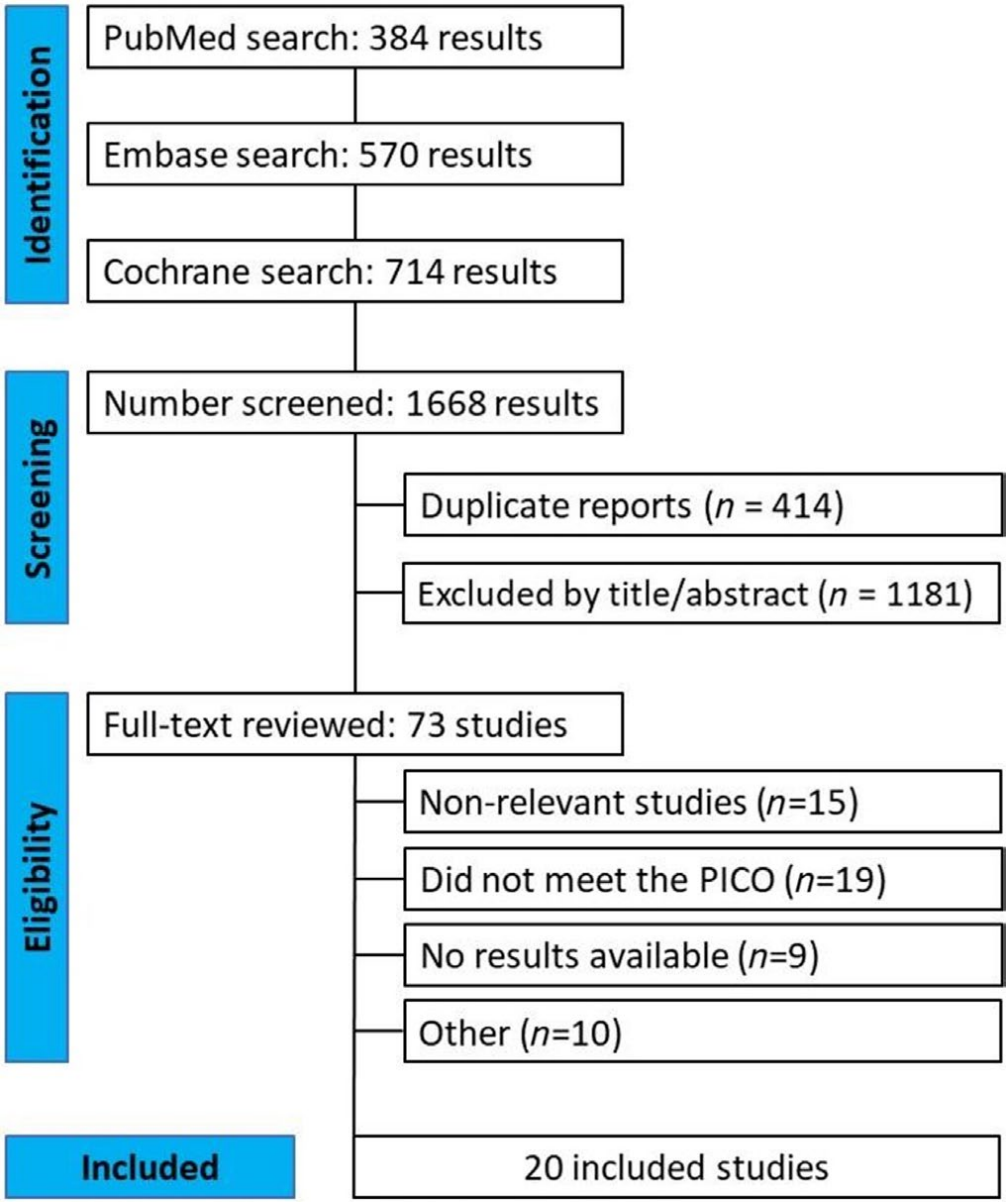
PRISMA flowchart for study screening and selection.

A PRISMA flow chart (Figure 1) shows the article selection process. The initial search identified 1668 results. Following duplicate removal and exclusion of ineligible studies based on predefined criteria, 73 articles underwent full‐text review. Of these, 20 studies were ultimately selected for inclusion in our meta‐analysis (detailed in Table 1) [23, 24, 25, 26, 27, 28, 29, 30, 31, 32, 33, 34, 35, 36, 37, 38, 39, 40, 41, 42]. The remaining 53 studies were excluded based on various factors: absence of results (*n* = 9), nonalignment with our PICO framework (*n* = 19), nonrelevant studies (*n* = 15), and other reasons (*n* = 9).

**TABLE 1:** Baseline characteristics of included studies (*n* = 20).

Study	Country	No. of patients	Age (years)^a^	Male (%)	ASA I or II (%)	Type of prosthesis		Last follow‐up (years)
		CH/UCH	CH/UCH	CH/UCH	CH/UCH	CH	UCH	
Abdelkhalek 2011	Egypt	15/10	NA	NA	NA	Thompson prosthesis	Austin Moore's prosthesis	6
Barenius 2018^(A)^	Sweden	39/44	NA	NA	NA	Exeter stem (Stryker, Kalamazoo, Michigan)	Hydroxyapatite‐coated bimetric stem (Zimmer Biomet, Warsaw, Indiana)	4
DeAngelis 2012	US	66/64	81.8/82.8	21.2/25	NA	Cemented VerSys LD/Fx (Zimmer)	Uncemented VerSys Beaded FullCoat (Zimmer)	1
Dorr 1986	US	37/13	72/66	30/30	NA	Bipolar cemented prosthesis	Bipolar uncemented prosthesis	2
Emery 1991	UK	27/26	78/79.6	11.11/15.38	NA	Bipolar prosthesis with a Thompson stem of the Monk duoplet design (Johnson & Johnson, England)	Bipolar prosthesis with a Moore stem of the Monk duoplet design (Johnson & Johnson, England)	2.5
Fernandez 2022	UK	610/615	84.5/84.3	31/33.2	NA	ODEP‐rated cemented femoral stem and head	Contemporary uncemented prosthesis (hydroxyapatite‐coated)	3
Figved 2009^(B)^	Norway	112/108	83.4/83	22/26	42/44	Bipolar prosthesis with cemented femoral stem (Spectron; Smith & Nephew Inc. Memphis, TN)	Bipolar prosthesis with uncemented femoral stem (Corail; DePuy International Ltd. Leeds, UK)	2
Inngul 2015^(A)^	Sweden	39/44	NA	NA	NA	Cemented Exeter stem (Stryker, Kalamazoo, Michigan) with a unipolar head	Cementless hydroxyapatite‐coated unipolar head	1
Langslet 2014^(B)^	Norway	112/108	83.4/83	22/26	42/44	Bipolar HA with cemented femoral stem (Spectron; Smith & Nephew Inc. Memphis, TN)	Bipolar HA with uncemented femoral stem (Corail; DePuy International Ltd. Leeds, UK)	5
Moerman 2017	Netherlands	110/91	83/84	25/33	71/64	Müller Straight Stem (Zimmer—Biomet, 1800 West Center St. Warsaw, Indiana, USA)	DB‐10 (Zimmer‐ Biomet, 1800 West Center St. Warsaw, Indiana, USA). HA‐coated	1
Movrin 2020	Slovenia	79/79	86/84	41.7/39.3	50.6/58	Ecofit prosthesis (80‐mg Palacos cement (Heraeus, Wehrheim, Germany)	Uncemented Ecofit prosthesis	2
Parker 2010	UK	200/200	83/83	20/27	NA	Thompson prosthesis (Corin Ltd. Cirencester, United Kingdom)	Austin‐Moore prosthesis (Stryker/Howmedica Ltd. Newbury, United Kingdom)	
Parker 2020^(C)^	UK	200/200	84.2/85.3	33.5/30	18/17.5	Unipolar, cemented polished double‐taper stem prosthesis	Hydroxyapatite‐coated uncemented Furlong prosthesis	1
Parker 2023^(C)^	UK	200/200	84.2/85.3	33.5/30	18/17.5	Unipolar, cemented polished double‐taper stem prosthesis	Hydroxyapatite‐coated uncemented Furlong prosthesis	3
Santini 2005	Italy	53/53	82.09/79.68	92.45/20.75	41.51/49.05	Bipolar cemented stem	Bipolar uncemented stem	1
Sonn‐Holm 1982	Denmark	40/35	NA	NA	NA	Cemented Moore prosthesis with methyl methacrylate	Uncemented Moore prosthesis	1
Talsnes 2013	Norway	162/172	84.3/84	27.6/21.5	42.2/39.7	Bipolar cemented prosthesis (Landos Titan, Depuy, Warshaw, IN, USA)	Bipolar uncemented implants (Landos Corail, Depuy, Warshaw, IN, USA)	1
Taylor 2012	New Zealand	80/80	85.3/85.1	28.75/33.75	NA	Exeter (Stryker, Kalamazoo, Michigan)	Zweymüller Alloclassic (Zimmer)	2
Vidovic 2013^(D)^	Croatia	30/30	82.9/82.04	0/0	NA	Cemented modular prosthesis	Uncemented modular Austin Moore prosthesis	1
Vidovic 2015^(D)^	Croatia	30/30	82.9/82.04	0/0	NA	Cemented modular prosthesis	Uncemented modular Austin Moore prosthesis	1

*Note*: Studies grouped with the same letter (A–D) represent the same trial but are cited separately due to differing follow‐up periods and unique data sets/outcomes.

Abbreviations: ASA, American Society of Anesthesiologists; CH, cemented hemiarthroplasty; UCH, uncemented hemiarthroplasty.

^a^ Mean age in years.

### 3.2 Characteristics of the Included Studies

Among the included studies, certain pairs of studies, namely, Barenius 2018 and Inguul 2015, Langslet 2014 and Figved 2009, Vidovic 2013 and Vidovic 2015, and Parker 2020 and Parker 2023, had the same patient cohorts but are included in Table 1 separately due to variations in follow‐up durations and outcome measures. From the included studies, the aggregate data comprised a total of 3680 patients, with 1860 (50.5%) undergoing cemented hemiarthroplasties and 1820 (49.5%) undergoing uncemented hemiarthroplasties. The geographical distribution of the studies was predominantly European, with 12 trials conducted in Europe, supplemented by 2 studies from North America, 1 from Africa, and 1 from Oceania. This wide temporal and geographical range, spanning from 1982 to 2023, mirrors the evolution of HA practices. The average age of participants was 82.35 years for those in the cemented group and 81.76 years for those in the uncemented group. Among these participants, 21.7% were male in the cemented group and 28.4% were male in the uncemented group.

### 3.3 Risk of Bias Assessment

**FIGURE 2:**
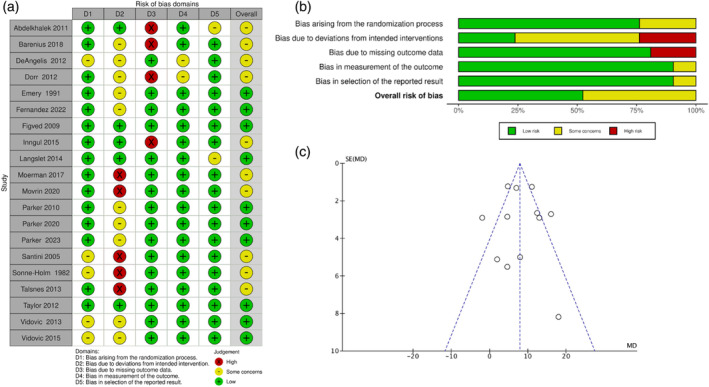
(a) Risks of bias of individual studies. (b) The sum of risk of bias assessment. (c) Funnel plot of the most reported outcome (length of surgery).

Of the 20 included RCTs [23, 24, 25, 26, 27, 28, 29, 30, 31, 32, 33, 34, 35, 36, 37, 38, 39, 40, 41, 42], 10 were assessed as having an overall moderate risk of bias [23, 24, 25, 26, 29, 31, 32, 36, 37, 38] and 10 were assessed as having a low risk of bias (Figure 2a) [27, 28, 30, 33, 34, 35, 39, 40, 41, 42]. The most common reason for bias was a deviation from the intended intervention (Figure 2b). A funnel plot of the most commonly reported outcome was generated to evaluate the risk of bias. The plot showed no asymmetry, indicating no risk of publication bias (Figure 2c). No relevant publication bias was detected using Egger's test (all *p*‐values > 0.05; range: 0.22–0.86).

### 3.4 Health‐Related Quality of Life (HRQoL)

**FIGURE 3:**
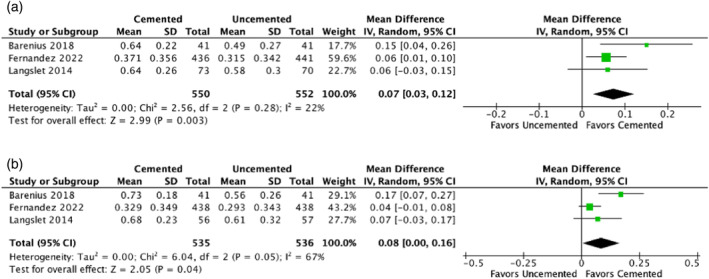
(a) Comparison of the EQ‐5D utility score at follow‐up after 3–4 months. (b) Comparison of the EQ‐5D utility score at follow‐up of 12 months. CI, confidence interval; IV, inverse variance.

In the early postoperative period (after 3–4 months), our analysis integrated data from three studies [24, 30, 42], encompassing a total of 550 who underwent cemented hemiarthroplasties and 552 patients who underwent uncemented procedures. The objective was to assess HRQoL using the EQ‐5D utility score. The MD in the EQ‐5D utility score was 0.07 (95% CI: 0.03–0.12). This difference was not only statistically significant (*p* = 0.003) but also demonstrated low heterogeneity among the studies (*I* ^2^ = 22%), as shown in Figure 3a. The direction of the effect indicates a higher score, favoring the cemented HA group.

At the 1‐year follow‐up, the EQ‐5D utility score was derived from three studies [24, 30, 42], involving a cohort of 535 patients who underwent cemented hemiarthroplasties and 536 patients with uncemented prostheses. We observed a significant MD of 0.08 (95% CI: 0.00–0.16) (*p* = 0.04). However, the heterogeneity among the studies was moderate (*I* ^2^ = 67%), as shown in Figure 3b.

### 3.5 Mortality

**FIGURE 4:**
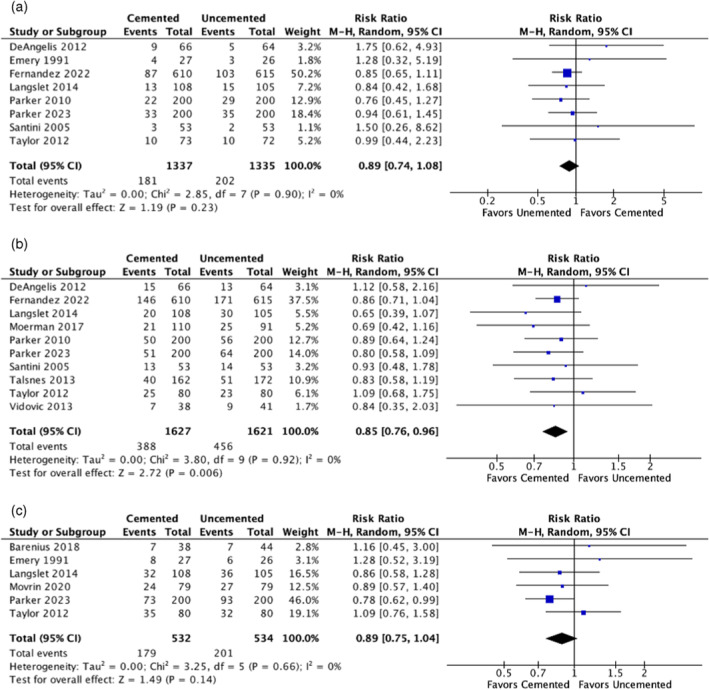
(a) Comparison of mortality rates at follow‐up of 4 months. Mantel–Haenszel. Comparison of mortality rates at follow‐up of 12 months. (c) Comparison of mortality rates at follow‐up of 2 years. CI, confidence interval; M–H, Mantel–Haenszel.

To assess short‐term mortality within the initial 4 months following surgery, we pooled data from eight studies [25, 27, 30, 34, 35, 36, 39, 42] involving 1337 patients with cemented hemiarthroplasties and 1335 patients with uncemented procedures. We determined an RR of 0.89 (95% CI: 0.74–1.08) with a *p*‐value of 0.23, suggesting no statistical significance. Notably, the analysis showed no heterogeneity among the studies included, as evidenced by an *I* ^2^ value of 0%, as shown in Figure 4a.

However, at the 1‐year postoperative milestone, mortality was evaluated across 10 studies [25, 30, 31, 34, 35, 36, 38, 39, 41, 42] (Figure 4b). The analysis included 1627 patients in the cemented group and 1621 in the uncemented group. The pooled RR was 0.85 (95% CI: 0.76–0.96) with a *p*‐value of 0.006, indicating a statistically significant reduction in mortality rates for the cemented HA group compared to the uncemented group. There was a complete absence of heterogeneity across the included studies, as reflected by an *I* ^2^ value of 0%.

Mortality after 2 years following surgery was assessed in six studies [24, 27, 30, 32, 35, 39], comprising 532 patients with cemented and 534 with uncemented hemiarthroplasties. The RR was 0.89 (95% CI: 0.75–1.04). There was no statistically significant difference in mortality rates (*p* = 0.14). Consistently, there was no evidence of heterogeneity among these studies (*I* ^2^ = 0%), as shown in Figure 4c.

### 3.6 Surgical‐Related Outcomes

The surgical‐related outcomes are shown in Table 2. Eleven studies [23, 25, 26, 30, 31, 32, 34, 35, 37, 39, 42] (1571 cemented patients and 1535 uncemented patients) showed a significant difference in the need for additional surgeries favoring the cemented group (MD: 0.69; 95% CI: 0.50–0.97; *p* = 0.03). No heterogeneity was observed (*I* ^2^ = 0%). Moreover, data from 12 studies [25, 27, 29, 30, 31, 32, 34, 35, 36, 38, 39, 41] (1158 cemented, 1147 uncemented) indicated that cemented procedures were significantly longer by an average of 8.04 min (MD: 8.04 min; 95% CI: 5.29–10.90; *p* < 0.00001), demonstrating high heterogeneity (*I* ^2^ = 75%). There was no significant difference in the duration of hospital stay, intraoperative blood loss, need for blood transfusion, or the number of units of transfused blood.

**TABLE 2:** The results of pooled analysis for surgical‐related outcomes.

Outcome	No. of studies	No. CH	No. UCH	Statistical method	Effect estimate	*p*	Favoring	Heterogeneity	
								*I* ^2^%	*p*
Surgical‐related outcomes									
Need for additional surgery	11	1571	1535	MD (IV, Random, 95% CI)	0.69 [0.50, 0.97]	0.03*	CH	0	0.94
Length of surgery	12	1158	1147	MD (IV, Random, 95% CI)	8.04 [5.29, 10.90]	< 0.00001*	UCH	75	< 0.00001
Duration of hospital stay	8	801	780	MD (IV, Random, 95% CI)	−0.31 [−1.01, 0.40]	0.40		0	0.74
Intraoperative blood loss	8	798	800	MD (IV, Random, 95% CI)	26.58 [−9.88, 63.05]	0.15		82	< 0.00001
Required for blood transfusion	6	1272	1257	MD (IV, Random, 95% CI)	1.00 [0.78, 1.29]	0.99		46	0.10
Units of blood transfused	3	453	453	MD (IV, Random, 95% CI)	−0.02 [−0.26, 0.22]	0.87		75	0.02

*Note*: Forest plots for the outcomes are in Supporting Information: Appendix S1.

Abbreviations: 95% CI, 95% confidence interval; CH, cemented hemiarthroplasty; MD, mean difference (for continuous outcomes); UCH, uncemented hemiarthroplasty.

* MD considered statistically significant (*p* < 0.05).

### 3.7 General Complications

In the analysis of general complications, as shown in Table 3, PE was reported as an outcome in seven studies [25, 27, 28, 31, 34, 35, 42] (1321 cemented, 1301 uncemented) demonstrated a significantly increased RR of 3.56 (95% CI: 1.26–10.11; *p* = 0.02), favoring uncemented HA. In addition, pressure sores/ulcers/decubitus, reported in five studies [27, 34, 35, 36, 42] (1091 cemented, 1094 uncemented), showed a significantly reduced RR of 0.58 (95% CI: 0.39–0.86; *p* = 0.007), favoring cemented HA. The incidence of pulmonary infections, cardiovascular events, DVT, cerebrovascular accidents, acute renal failure, and UTI did not significantly differ between the two arms of our analysis. Our pooled data showed no significant heterogeneity across the studies for any general complications.

**TABLE 3:** The results of pooled analysis for general and local complications.

Outcome	No. of studies	No. CH	No. UCH	Statistical method	Effect estimate	*p*	Favoring	Heterogeneity	
								*I* ^2^%	*p*
General complications									
Pulmonary infections	8	1405	1384	RR (M–H, Random, 95% CI)	0.78 [0.50, 1.21]	0.27		0	0.57
Cardiovascular events	8	1427	1408	RR (M–H, Random, 95% CI)	1.08 [0.64, 1.84]	0.77		0	0.94
DVT	6	1294	1275	RR (M–H, Random, 95% CI)	1.18 [0.51, 2.71]	0.70		0	0.85
PE	7	1321	1301	RR (M–H, Random, 95% CI)	3.56 [1.26, 10.11]	0.02*	UCH	0	0.98
Pressure sores/ulcer/decubitus	5	1091	1094	RR (M–H, Random, 95% CI)	0.58 [0.39, 0.86]	0.007*	CH	0	0.67
Cerebrovascular accident	5	1186	1170	RR (M–H, Random, 95% CI)	0.93 [0.41, 2.10]	0.86		0	0.57
Acute renal failure	4	1120	1106	RR (M–H, Random, 95% CI)	1.23 [0.76, 2.00]	0.39		0	0.59
UTI	5	880	865	RR (M–H, Random, 95% CI)	0.89 [0.65, 1.20]	0.43		0	0.64
Local complications									
Dislocation	9	1466	1454	RR (M–H, Random, 95% CI)	1.06 [0.58, 1.96]	0.84		0	0.84
Intraoperative fracture	8	900	875	RR (M–H, Random, 95% CI)	0.20 [0.09, 0.43]	< 0.0001*	CH	0	0.48
Periprosthetic/postoperative fracture	7	1391	1373	RR (M–H, Random, 95% CI)	0.18 [0.10, 0.33]	< 0.0001*	CH	0	0.99
Wound hematoma	3	510	491	RR (M–H, Random, 95% CI)	1.84 [0.60, 5.62]	0.29		0	0.48
Superficial infection	10	1475	1449	RR (M–H, Random, 95% CI)	1.16 [0.69, 1.96]	0.58		0	0.85
Deep infection	8	874	864	RR (M–H, Random, 95% CI)	2.09 [0.95, 4.59]	0.07		0	0.95

*Note*: Forest plots for the outcomes are in Supporting Information: Appendix S1.

Abbreviations: 95% CI, 95% confidence interval; CH, cemented hemiarthroplasty; DVT, deep vein thrombosis; PE, pulmonary embolism; RR, risk ratio (for dichotomous outcomes); UCH, uncemented hemiarthroplasty; UTI, urinary tract infection.

* RR considered statistically significant (*p* < 0.05).

### 3.8 Local Complications

In the evaluation of local complications, as shown in Table 3, our analysis revealed a significantly lower risk of intraoperative fractures in the cemented group, as seen in eight studies [25, 28, 31, 32, 34, 35, 36, 39] (900 cemented, 875 uncemented), with a risk ratio of 0.20 (95% CI: 0.09–0.43; *p* < 0.0001). Similarly, the risk of periprosthetic or postoperative fractures was significantly lower in the cemented group, as shown in seven studies [28, 31, 32, 34, 35, 39, 42] (1391 cemented, 1373 uncemented), with an RR of 0.18 (95% CI: 0.10–0.33; *p* < 0.00001). The risk of dislocation, wound hematoma, superficial infections, and deep infections did not differ significantly between the groups. There was no significant heterogeneity in any of the outcomes of local complications.

## 4 Discussion

Quality of life is paramount in older patients when evaluating surgical outcomes and medical interventions. Measuring clinical effectiveness is crucial, but it is only half the story. Research must also consider how treatments influence patients' day‐to‐day experiences including emotional well‐being, symptomatology, and daily motivation. This ensures that interventions extend life and improve quality of life, making patient‐centered outcomes a critical aspect of surgical decision‐making.

To the best of our knowledge, this is the first meta‐analysis to comprehensively explore the differences in quality of life between patients who underwent cemented versus uncemented HA. Our analysis revealed that cemented HA was associated with significantly better HRQoL at 3–4 months and 12 months post‐surgery, as measured by the EQ‐5D. These findings align with the most recently published RCT by Fernandez et al. (2022) [42], which also noted superior short and long‐term functional outcomes for cemented stems. However, our results diverged from those of the most recent meta‐analysis by Fu et al. (2021) [43], who did not distinguish between total hip and HA when examining EQ‐5D outcomes and reported no significant difference between cemented and uncemented arthroplasty in general [43].

In terms of mortality, our study revealed no significant differences between the cemented and uncemented groups within 4 months and at 2 years post operation, which is consistent with previous meta‐analyses [11, 18, 44, 45, 46]. However, our analysis revealed a significantly lower mortality rate in the cemented group at the one‐year follow‐up, underscoring the potential advantages of cemented approaches in enhancing survival rates within the first year following surgery. This finding contrasts with most recent reviews that suggested no detrimental effect at 1 year [11, 18, 44, 46]. One recent study showed the same significance at 1 year [45].

Similar to previous meta‐analyses, our study showed a significantly increased length of surgery in the cemented group. This can be attributed to the additional steps required for cement preparation and application [15, 17, 19, 44, 45, 46]. Length of hospital stay, intraoperative blood loss, need for blood transfusions, and number of units of blood transferred were not significantly different between the two groups. However, the need for additional surgeries was significantly lower in the cemented group. Longer surgeries in the cemented group are a potential drawback, but the prospect of a better quality of life and avoiding future procedures might outweigh this concern for some patients.

General complications are often considered a downfall for cemented HA [14]. Our analysis of general and local complications associated with cemented versus uncemented HA demonstrated that the incidence of general complications, including pulmonary infections, cardiovascular events, DVT, and UTIs, did not differ significantly between the two groups. This suggests that the choice of cementing does not substantially affect the likelihood of these systemic health issues postoperatively. Interestingly, PE showed increased risk in the cemented group, while pressure sores/ulcers/decubitus were significantly less common in the cemented group. This may be caused by the better mobility of patients who underwent cemented HA during the postoperative period. Recently published meta‐analyses showed similar results [11, 15, 19, 44, 45, 46].

Regarding local complications, our study revealed no significant differences in the risk of dislocation, wound hematoma, or superficial and deep infections between the two groups. However, a significant finding was the lower risk of intraoperative, periprosthetic/postoperative fractures in the cemented group. This finding makes cemented HA a more stable and durable solution, potentially reducing the risk of subsequent surgeries and improving overall health. This result was shown in multiple previous meta‐analyses showing the upper hand of cemented hemiarthroplasties in lowering the risk of operative fractures [11, 15, 17, 44, 46].

This study has several limitations. First, we included only studies published in English. Additionally, several studies had small sample sizes, which increases the risk of bias. Furthermore, we did not exclude studies based on criteria such as implant type (unipolar or bipolar), type of stem, or surgical approach (anterior or posterior). We also did not restrict the study to the type of cement used or the coating in uncemented implants. A more detailed comparison of EQ‐5D scores, accounting for factors such as surgical techniques, implant types, and subgroup analyses by age and gender, will require additional studies and data. We intend to explore these aspects in future research. Moreover, we observed a noticeable dropout rate in many of the included studies, likely attributable to the advanced age of the population. This dropout could have influenced our findings, suggesting that more consistent follow‐up might have led to different outcomes. In addition, we included elderly and cognitively impaired patients, and data were reported by proxy rather than from the patients themselves in some of the studies, potentially causing bias.

The choice between cemented and uncemented HA for treating FNFs in elderly patients requires careful consideration of individual risk factors and the potential advantages and disadvantages of each technique. While both procedures offer effective solutions, cemented HA may offer better HRQoL. On the other hand, uncemented procedures might be associated with lower mortality at 1 year. From a policy perspective, our results reinforce the need for guidelines that advocate for patient‐centered care approaches, considering both the physiological and psychosocial aspects of recovery from hip fractures.

## Author Contributions

All authors had full access to the data in the study and took responsibility for the integrity of the data and the accuracy of the data analysis. Conceptualization, Mohanad Samaheen; methodology, Mohanad Samaheen and Maen Mohammad; investigation, Mohanad Samaheen and Maen Mohammad; formal analysis, Mohanad Samaheen, Maen Mohammad, and Mikhail Salzmann; resources, Mohanad Samaheen and Maen Mohammad; writing – original draft, Mohanad Samaheen and Maen Mohammad; writing – review and editing, Mohanad Samaheen, Maen Mohammad, and Nikolai Ramadanov; visualization, Mohanad Samaheen; supervision, Nikolai Ramadanov; funding acquisition, Maen Mohammad.

## Disclosure

All authors take responsibility for the integrity of the data and the accuracy of the analysis. No external funding was received for this study.

## Conflicts of Interest

The authors declare no conflicts of interest.

## Supporting information


Appendix S1.

## Data Availability

The data that support the findings of this study are available from the corresponding author upon reasonable request.
