# Supplementary material for: The Health‐Related Quality of Life for Cemented Versus Uncemented Hemiarthroplasty in Elderly Patients With Femoral Neck Fractures: A Systematic Review and Meta‐Analysis of Randomized Controlled Trials
Source: Orthop Surg. 2024 Dec 26;17(2):361–72. doi: 10.1111/os.14339 (PMC11787973; doi:10.1111/os.14339)
Supplement: Supplementary file 1 — Appendix S1. [file OS-17-361-s001.docx]

**Supplementary Appendix**

This appendix has been provided by the authors to give readers additional information about the work.

Table of Contents

[Search strategy: 3](#_Toc162567540)

[Figure (1): Forest plot for comparison of need for additional surgery between the cemented hemiarthroplasty group and uncemented hemiarthroplasty group. 4](#_Toc162567541)

[Figure (2): Forest plot for comparison of length of surgery between the cemented hemiarthroplasty group and uncemented hemiarthroplasty group. 4](#_Toc162567542)

[Figure (3): Forest plot for comparison of duration of hospital stay between the cemented hemiarthroplasty group and uncemented hemiarthroplasty group. 5](#_Toc162567543)

[Figure (4): Forest plot for comparison of intraoperative blood loss between the cemented hemiarthroplasty group and uncemented hemiarthroplasty group. 5](#_Toc162567544)

[Figure (5): Forest plot for comparison of requirement of blood transfusion between the cemented hemiarthroplasty group and uncemented hemiarthroplasty group. 5](#_Toc162567545)

[Figure (6): Forest plot for comparison of units of blood transfused between the cemented hemiarthroplasty group and uncemented hemiarthroplasty group. 6](#_Toc162567546)

[Figure (7): Forest plot for comparison of pulmonary infections between the cemented hemiarthroplasty group and uncemented hemiarthroplasty group. 6](#_Toc162567547)

[Figure (8): Forest plot for comparison of cardiovascular events between the cemented hemiarthroplasty group and uncemented hemiarthroplasty group. 7](#_Toc162567548)

[Figure (9): Forest plot for comparison of DVT between the cemented hemiarthroplasty group and uncemented hemiarthroplasty group. 7](#_Toc162567549)

[Figure (10): Forest plot for comparison of PE between the cemented hemiarthroplasty group and uncemented hemiarthroplasty group. 7](#_Toc162567550)

[Figure (11): Forest plot for comparison of pressure sores/ulcer/decubitus between the cemented hemiarthroplasty group and uncemented hemiarthroplasty group. 8](#_Toc162567551)

[Figure (12): Forest plot for comparison of cerebrovascular accident between the cemented hemiarthroplasty group and uncemented hemiarthroplasty group. 8](#_Toc162567552)

[Figure (13): Forest plot for comparison of acute renal failure between the cemented hemiarthroplasty group and uncemented hemiarthroplasty group. 8](#_Toc162567553)

[Figure (14): Forest plot for comparison of UTI between the cemented hemiarthroplasty group and uncemented hemiarthroplasty group. 9](#_Toc162567554)

[Figure (15): Forest plot for comparison of dislocation between the cemented hemiarthroplasty group and uncemented hemiarthroplasty group. 9](#_Toc162567555)

[Figure (16): Forest plot for comparison of intraoperative fracture between the cemented hemiarthroplasty group and uncemented hemiarthroplasty group. 10](#_Toc162567556)

[Figure (18): Forest plot for comparison of wound hematoma between the cemented hemiarthroplasty group and uncemented hemiarthroplasty group. 10](#_Toc162567557)

[Figure (19): Forest plot for comparison of superficial infection between the cemented hemiarthroplasty group and uncemented hemiarthroplasty group. 11](#_Toc162567558)

[Figure (20): Forest plot for comparison of deep infection between the cemented hemiarthroplasty group and uncemented hemiarthroplasty group. 11](#_Toc162567559)

## **Search strategy:**

("hemiarthroplasty"[MeSH Terms] OR "hemiarthroplasty"[All Fields] OR "hemiarthroplasties"[All Fields] OR ("hemiarthroplasty"[MeSH Terms] OR "hemiarthroplasty"[All Fields] OR "hemiarthroplasties"[All Fields]) OR ("hemiarthroplasty"[MeSH Terms] OR "hemiarthroplasty"[All Fields] OR ("hemi"[All Fields] AND "arthroplasty"[All Fields]) OR "hemi arthroplasty"[All Fields]) OR ("hemiarthroplasty"[MeSH Terms] OR "hemiarthroplasty"[All Fields] OR ("hemi"[All Fields] AND "arthroplasty"[All Fields]) OR "hemi arthroplasty"[All Fields]) OR ("hemiarthroplasty"[MeSH Terms] OR "hemiarthroplasty"[All Fields] OR ("hemi"[All Fields] AND "arthroplasties"[All Fields]) OR "hemi arthroplasties"[All Fields])) AND ("cement s"[All Fields] OR "cementable"[All Fields] OR "cementation"[MeSH Terms] OR "cementation"[All Fields] OR "cementations"[All Fields] OR "cementing"[All Fields] OR "dental cementum"[MeSH Terms] OR ("dental"[All Fields] AND "cementum"[All Fields]) OR "dental cementum"[All Fields] OR "cement"[All Fields] OR "dental cements"[MeSH Terms] OR ("dental"[All Fields] AND "cements"[All Fields]) OR "dental cements"[All Fields] OR "cemented"[All Fields] OR "cements"[All Fields] OR ("cement s"[All Fields] OR "cementable"[All Fields] OR "cementation"[MeSH Terms] OR "cementation"[All Fields] OR "cementations"[All Fields] OR "cementing"[All Fields] OR "dental cementum"[MeSH Terms] OR ("dental"[All Fields] AND "cementum"[All Fields]) OR "dental cementum"[All Fields] OR "cement"[All Fields] OR "dental cements"[MeSH Terms] OR ("dental"[All Fields] AND "cements"[All Fields]) OR "dental cements"[All Fields] OR "cemented"[All Fields] OR "cements"[All Fields]) OR ("cement s"[All Fields] OR "cementable"[All Fields] OR "cementation"[MeSH Terms] OR "cementation"[All Fields] OR "cementations"[All Fields] OR "cementing"[All Fields] OR "dental cementum"[MeSH Terms] OR ("dental"[All Fields] AND "cementum"[All Fields]) OR "dental cementum"[All Fields] OR "cement"[All Fields] OR "dental cements"[MeSH Terms] OR ("dental"[All Fields] AND "cements"[All Fields]) OR "dental cements"[All Fields] OR "cemented"[All Fields] OR "cements"[All Fields]) OR "cement*"[All Fields]) AND ("uncement"[All Fields] OR "uncemented"[All Fields] OR "uncement*"[All Fields] OR "cementless"[All Fields] OR "non cement*"[All Fields] OR "noncement"[All Fields] OR "hydroxyapatite-coated"[All Fields]) AND ("hip"[MeSH Terms] OR "hip"[All Fields] OR ("femur"[MeSH Terms] OR "femur"[All Fields] OR "femurs"[All Fields] OR "femur s"[All Fields] OR "femural"[All Fields] OR "femure"[All Fields]) OR ("femor"[All Fields] OR "femorals"[All Fields] OR "femur"[MeSH Terms] OR "femur"[All Fields] OR "femoral"[All Fields]) OR ("hip fractures"[MeSH Terms] OR ("hip"[All Fields] AND "fractures"[All Fields]) OR "hip fractures"[All Fields]))


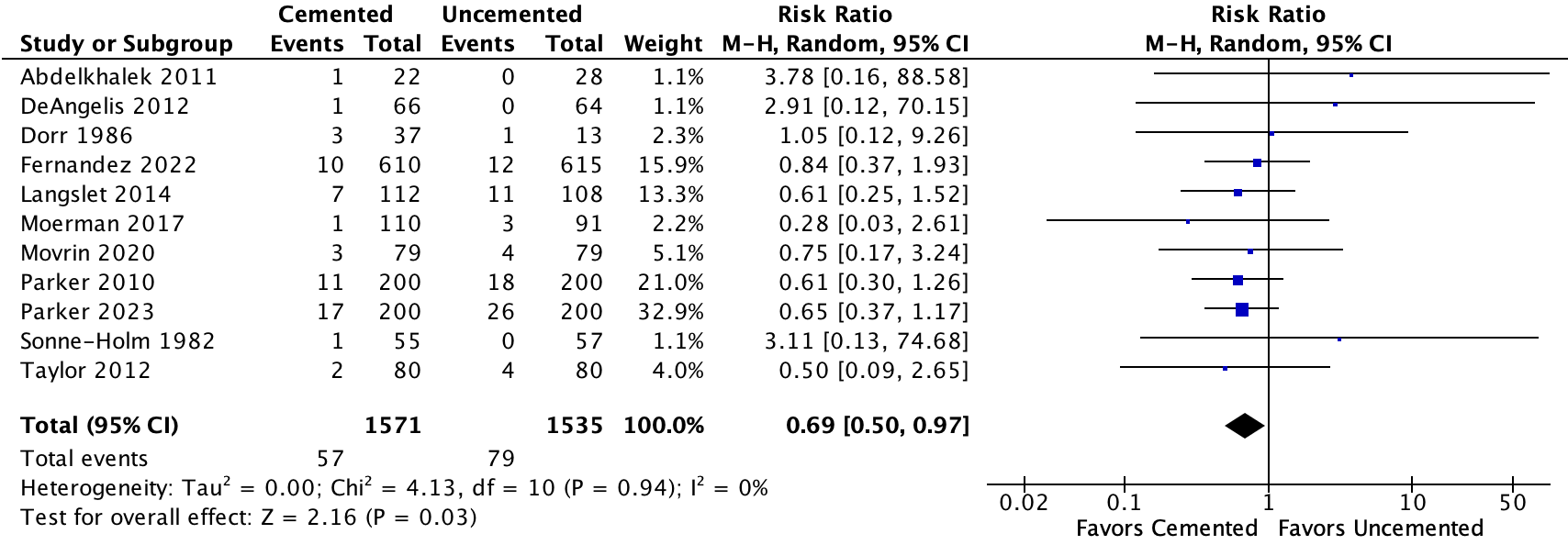
Figure (1): Forest plot for comparison of need for additional surgery between the cemented hemiarthroplasty group and uncemented hemiarthroplasty group.


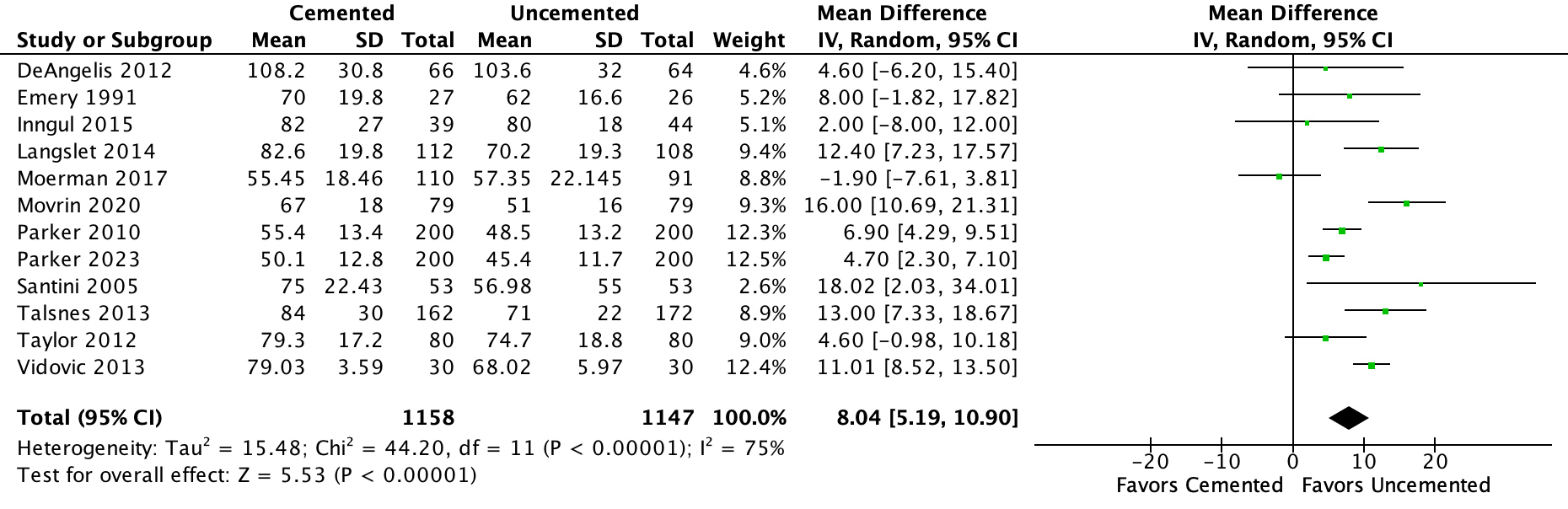
Figure (2): Forest plot for comparison of length of surgery between the cemented hemiarthroplasty group and uncemented hemiarthroplasty group.


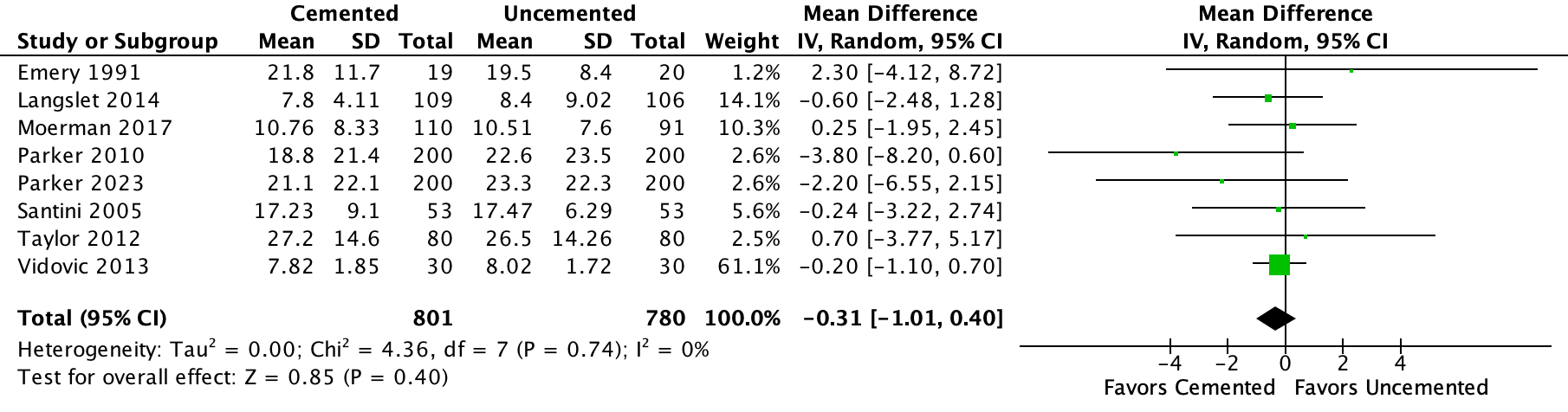


## Figure (3): Forest plot for comparison of duration of hospital stay between the cemented hemiarthroplasty group and uncemented hemiarthroplasty group.


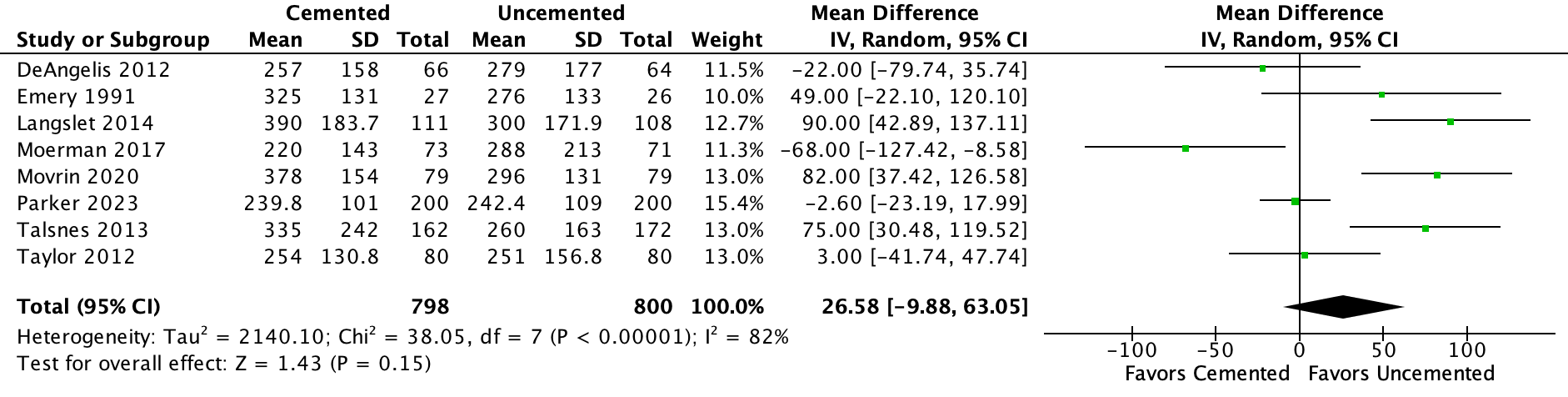


## Figure (4): Forest plot for comparison of intraoperative blood loss between the cemented hemiarthroplasty group and uncemented hemiarthroplasty group.


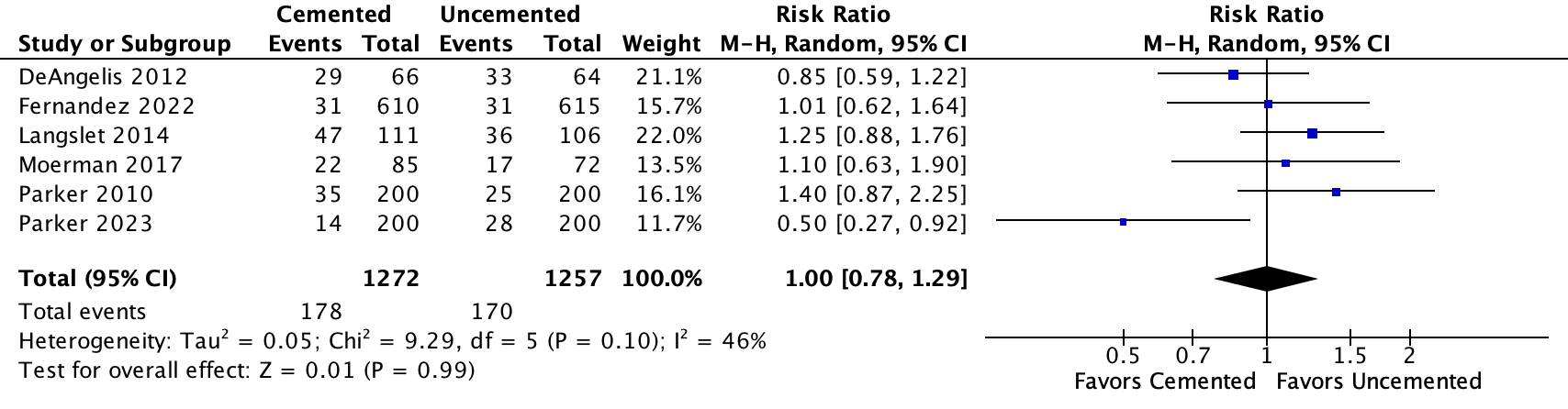


## Figure (5): Forest plot for comparison of requirement of blood transfusion between the cemented hemiarthroplasty group and uncemented hemiarthroplasty group.


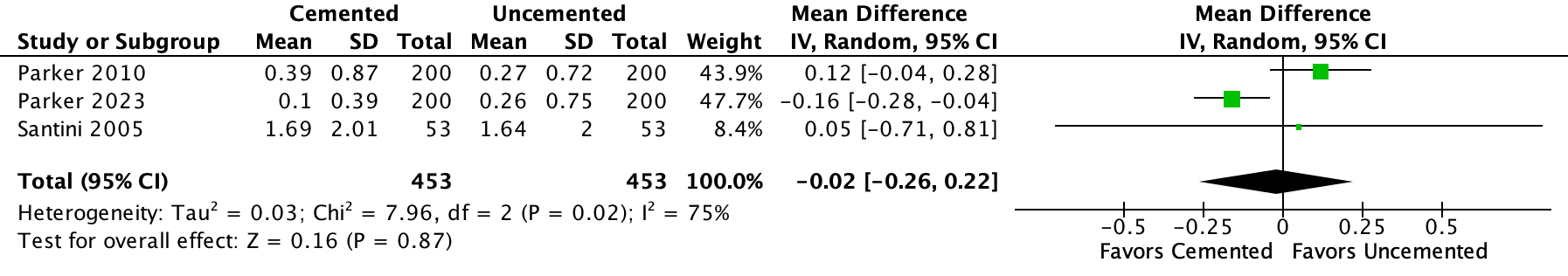


## Figure (6): Forest plot for comparison of units of blood transfused between the cemented hemiarthroplasty group and uncemented hemiarthroplasty group.


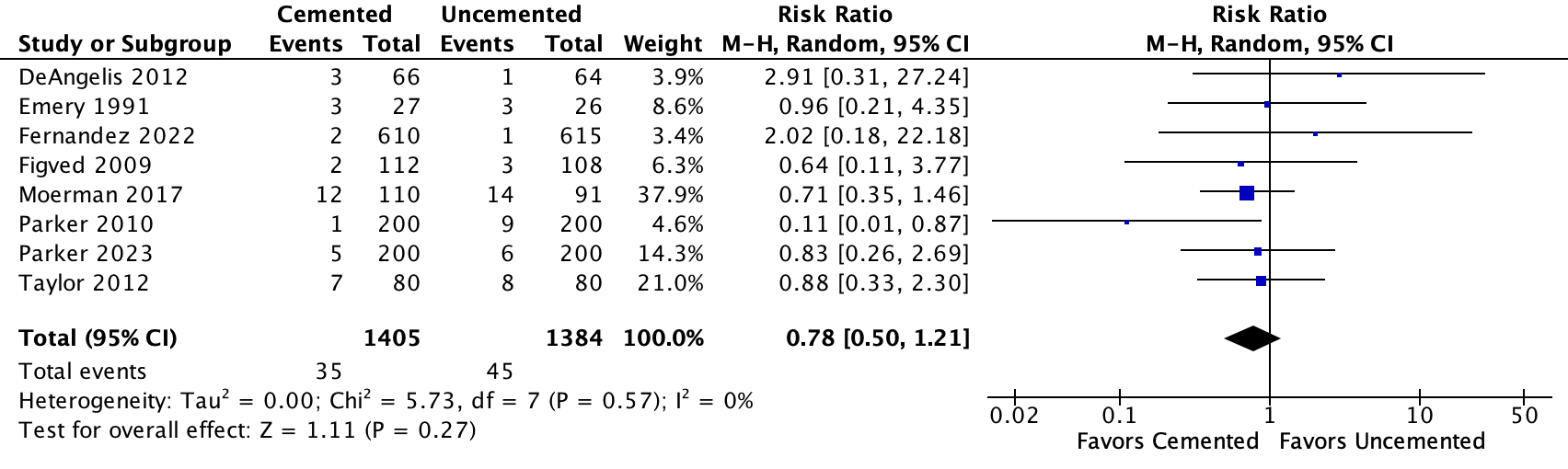


## Figure (7): Forest plot for comparison of pulmonary infections between the cemented hemiarthroplasty group and uncemented hemiarthroplasty group.


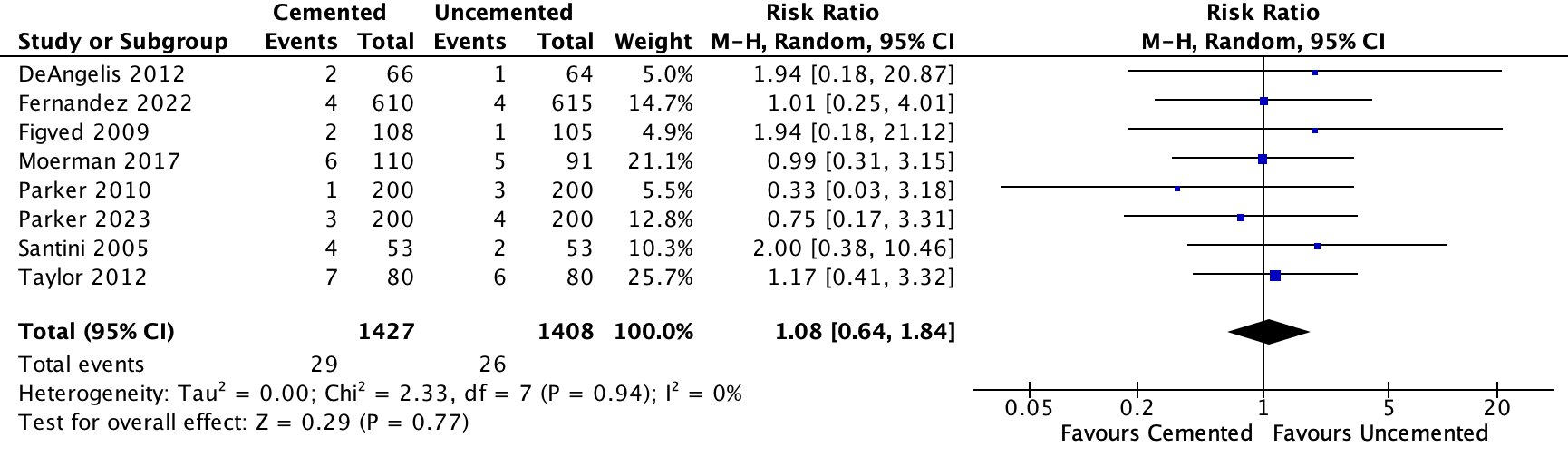


## Figure (8): Forest plot for comparison of cardiovascular events between the cemented hemiarthroplasty group and uncemented hemiarthroplasty group.


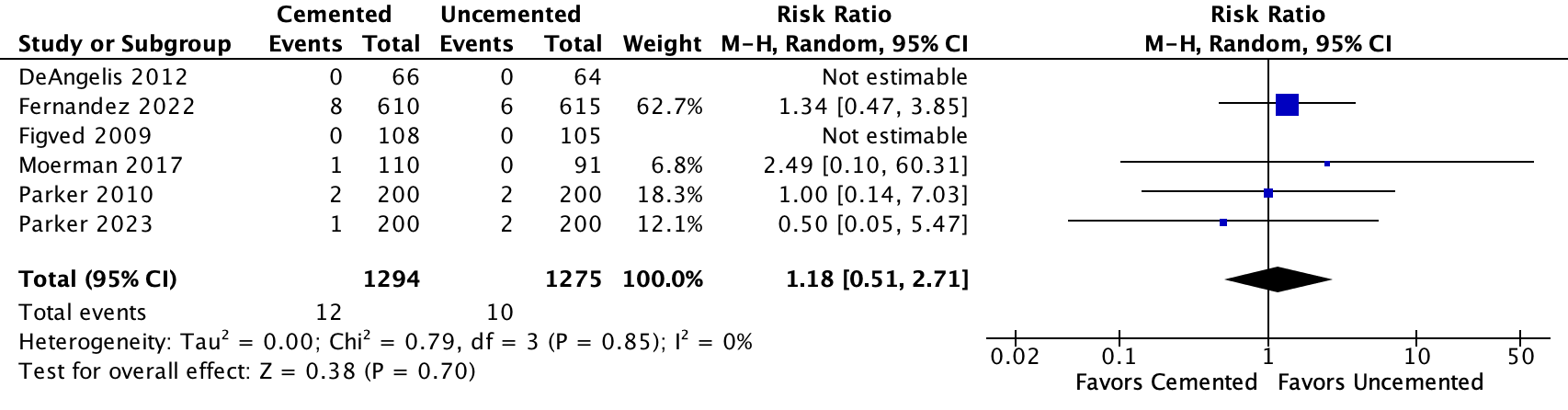


## Figure (9): Forest plot for comparison of DVT between the cemented hemiarthroplasty group and uncemented hemiarthroplasty group.


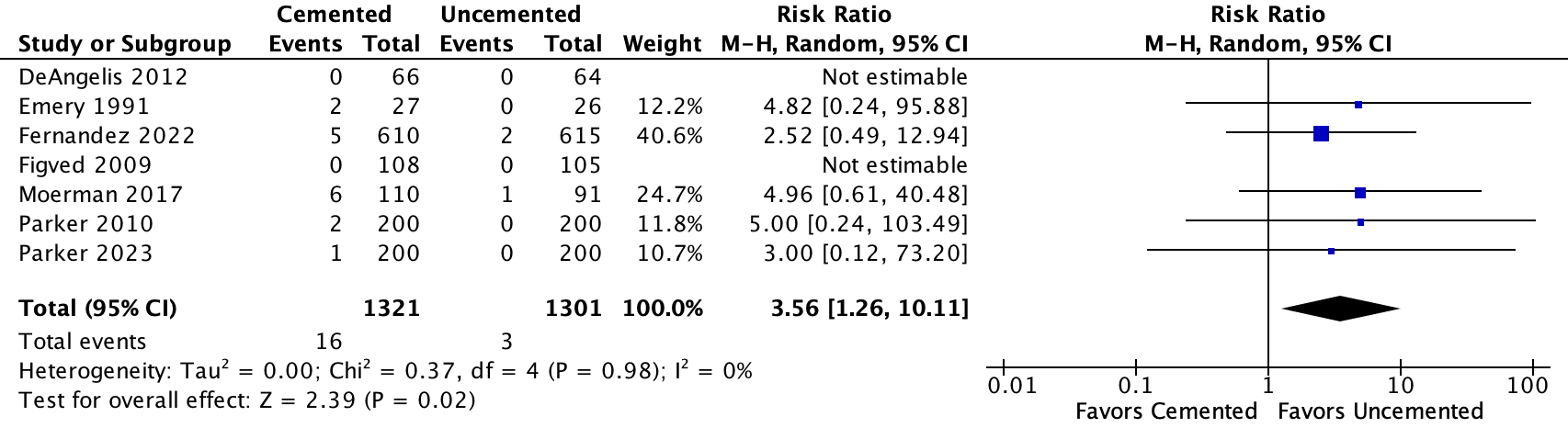


## Figure (10): Forest plot for comparison of PE between the cemented hemiarthroplasty group and uncemented hemiarthroplasty group.


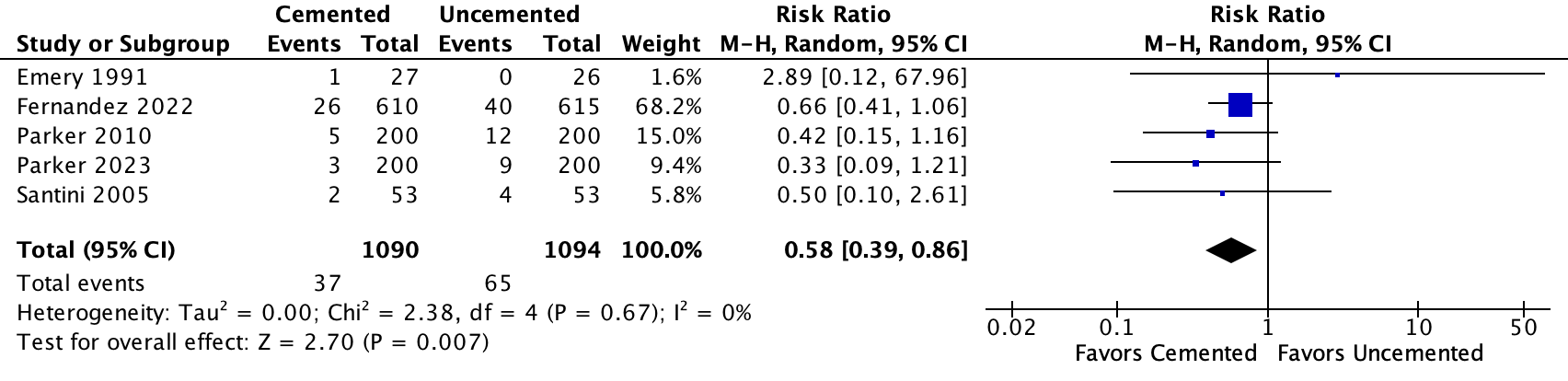


## Figure (11): Forest plot for comparison of pressure sores/ulcer/decubitus between the cemented hemiarthroplasty group and uncemented hemiarthroplasty group.


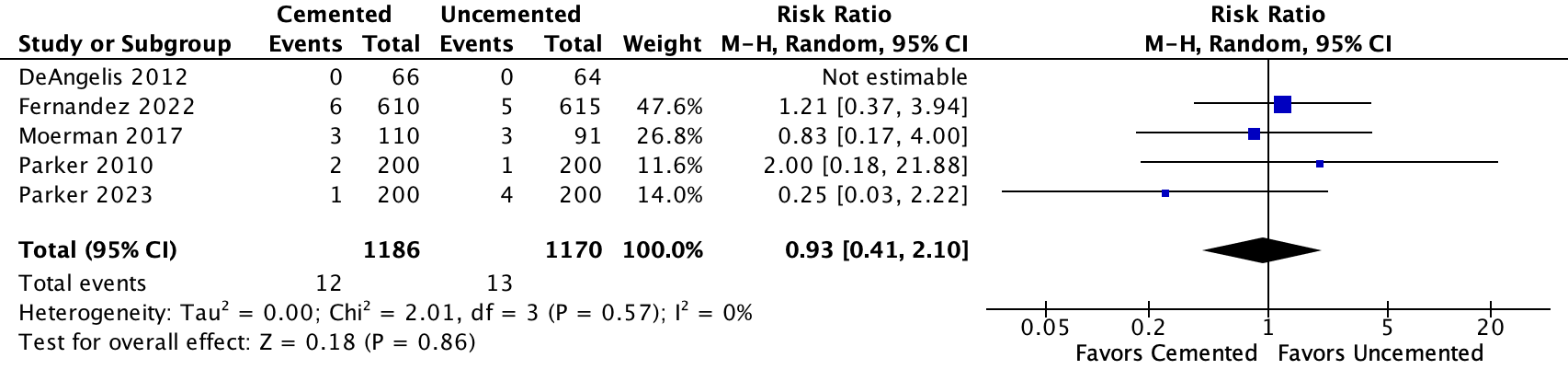


## Figure (12): Forest plot for comparison of cerebrovascular accident between the cemented hemiarthroplasty group and uncemented hemiarthroplasty group.


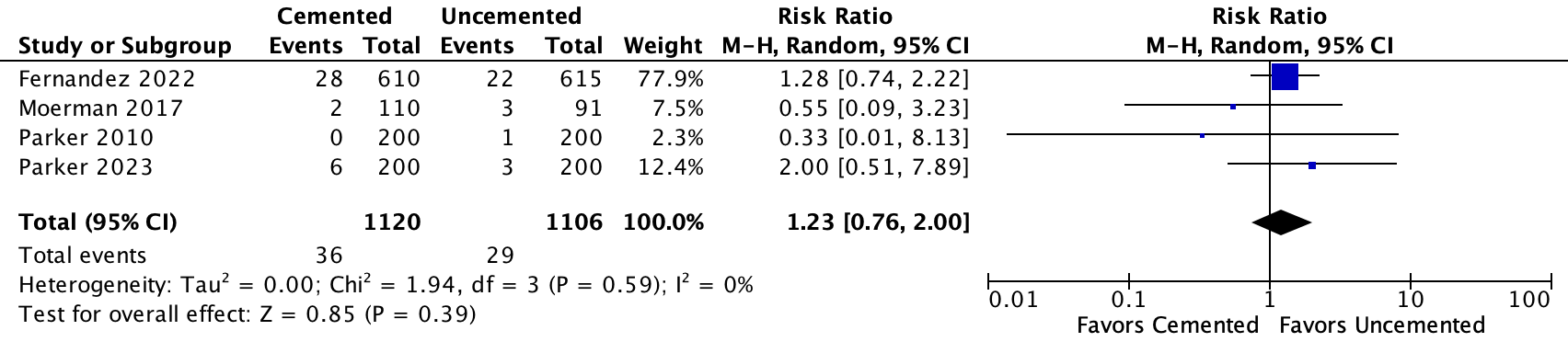


## Figure (13): Forest plot for comparison of acute renal failure between the cemented hemiarthroplasty group and uncemented hemiarthroplasty group.


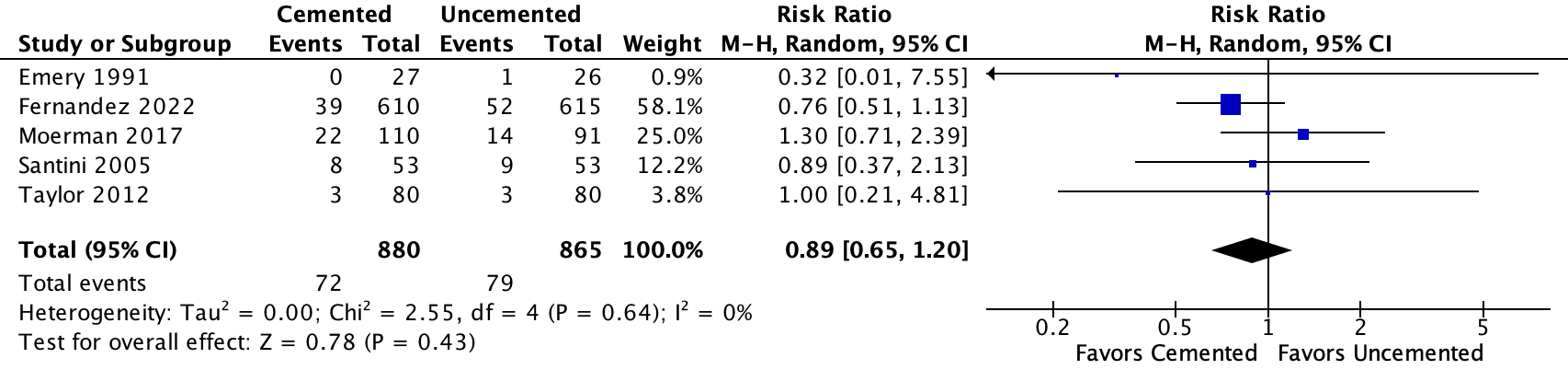


## Figure (14): Forest plot for comparison of UTI between the cemented hemiarthroplasty group and uncemented hemiarthroplasty group.


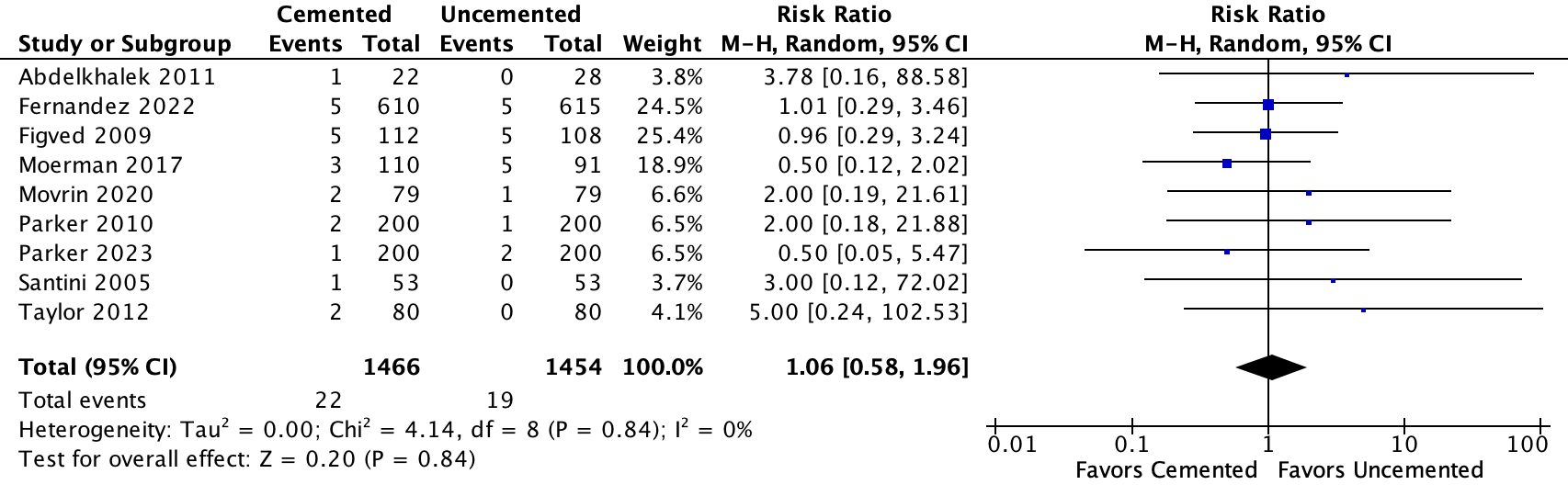


## Figure (15): Forest plot for comparison of dislocation between the cemented hemiarthroplasty group and uncemented hemiarthroplasty group.


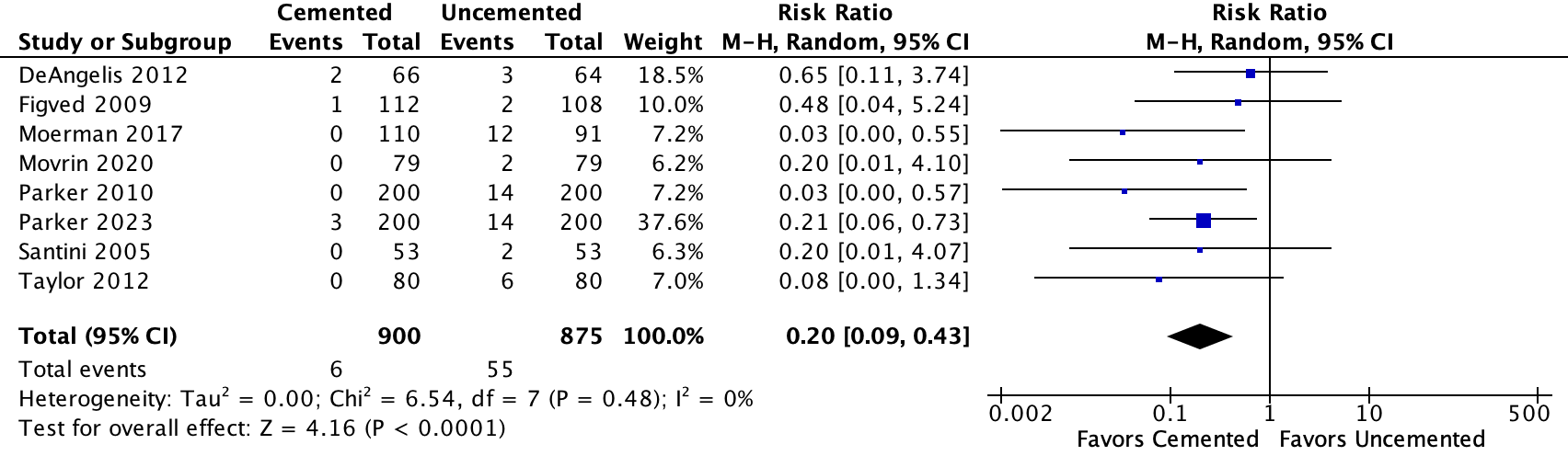


## Figure (16): Forest plot for comparison of intraoperative fracture between the cemented hemiarthroplasty group and uncemented hemiarthroplasty group.


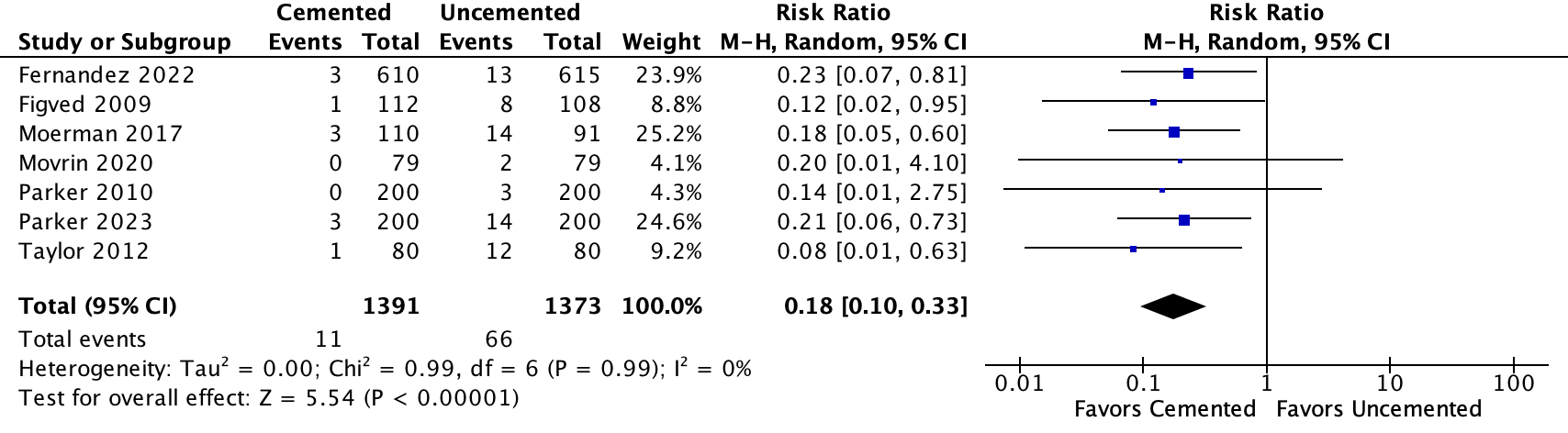


Figure (17): Forest plot for comparison of periprosthetic/postoperative fracture between the cemented hemiarthroplasty group and uncemented hemiarthroplasty group.


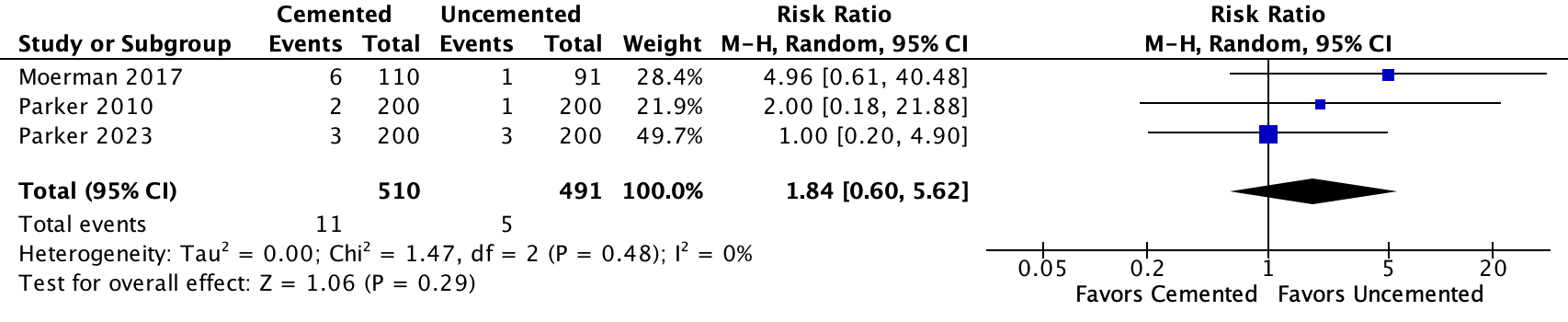


## Figure (18): Forest plot for comparison of wound hematoma between the cemented hemiarthroplasty group and uncemented hemiarthroplasty group.


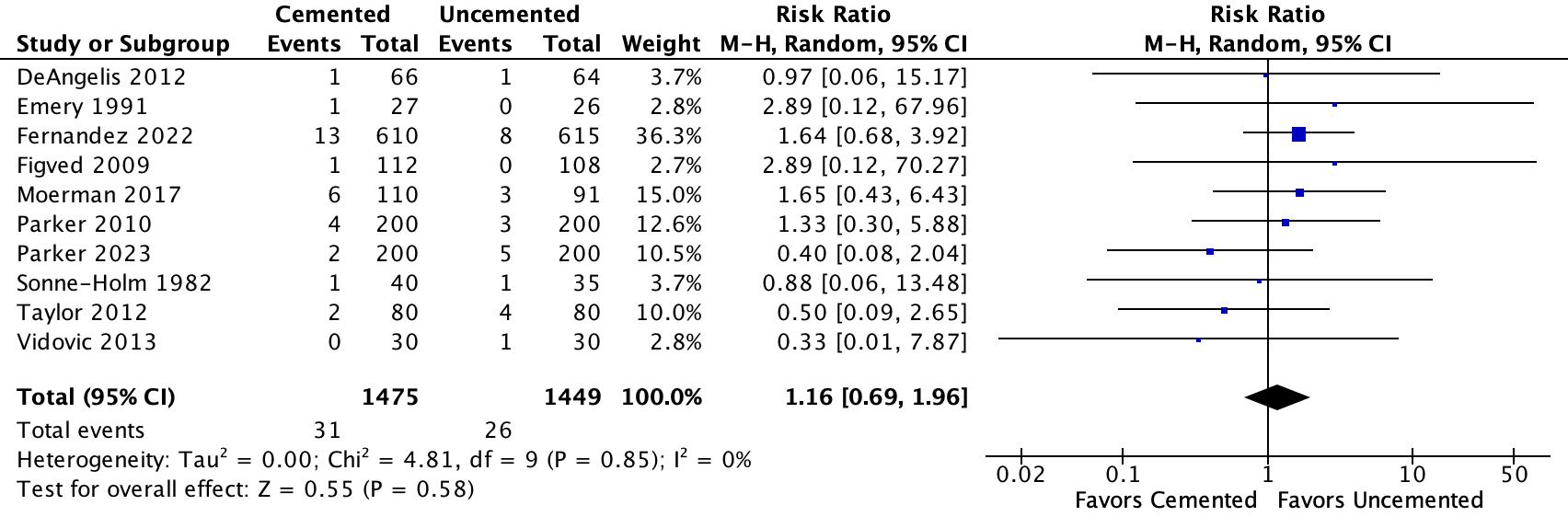


## Figure (19): Forest plot for comparison of superficial infection between the cemented hemiarthroplasty group and uncemented hemiarthroplasty group.


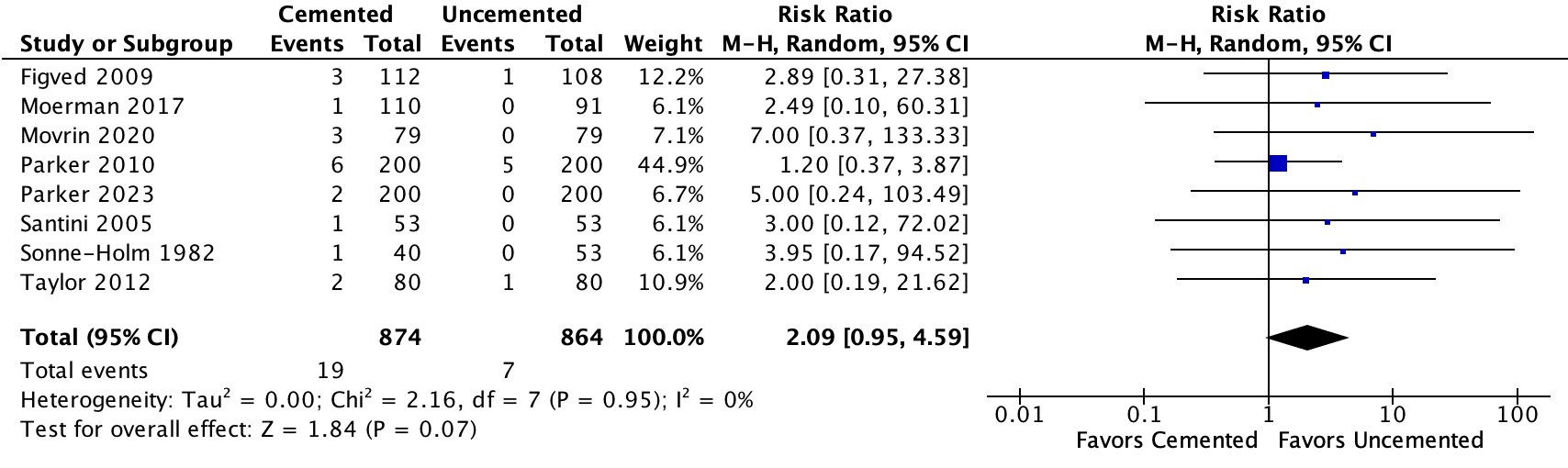


## Figure (20): Forest plot for comparison of deep infection between the cemented hemiarthroplasty group and uncemented hemiarthroplasty group.
